# Could Vitamin D Analogues Be Used to Target Leukemia Stem Cells?

**DOI:** 10.3390/ijms17060889

**Published:** 2016-06-06

**Authors:** Idoia García-Ramírez, Alberto Martín-Lorenzo, Inés González-Herrero, Guillermo Rodriguez-Hernández, Carolina Vicente-Dueñas, Isidro Sánchez-García

**Affiliations:** 1 Experimental Therapeutics and Translational Oncology Program, Instituto de Biología Molecular y Celular del Cáncer, CSIC/Universidad de Salamanca, Campus M. de Unamuno s/n, Salamanca 37007, Spain; idoia.g@usal.es (I.G.-R.); trioney@usal.es (A.M.-L.); ighe@usal.es (I.G.-H.); guillermorh@usal.es (G.R.-H.); 2 Cancer Research Area, Institute of Biomedical Research of Salamanca (IBSAL), Salamanca 37007, Spain

**Keywords:** cancer, stem cells, vitamin D, cancer stem cells, mouse models, leukemia therapy

## Abstract

Leukemic stem cells (LSCs) are defined as cells that possess the ability to self-renew and give rise to the differentiated cancer cells that comprise the tumor. These LSCs seem to show chemo-resistance and radio-resistance leading to the failure of conventional cancer therapies. Current therapies are directed at the fast growing tumor mass leaving the LSC fraction untouched. Eliminating LSCs, the root of cancer origin and recurrence, is considered to be a hopeful approach to improve survival or even to cure cancer patients. In order to achieve this, the characterization of LSCs is a prerequisite in order to develop LSC-based therapies to eliminate them. Here we review if vitamin D analogues may allow an avenue to target the LSCs.

## 1. Introduction

Cancer treatment has not significantly changed during recent decades despite our increased knowledge of the biology of cancer cells [1]. Survival rates for the main types of cancer have changed very little in the last few decades once cancer is disseminated. On the contrary, survival is relatively good when cancer is diagnosed at the early stage. This observation implies that the improvement in global cancer survival is mostly the consequence of early detection rather than the result of an effective therapy once the tumor has extended. Thus, questions remain open as to whether current cancer compounds target the wrong kind of cells or cancer cell biological properties.

An emerging topic in cancer biology has been the existence of a “cancer stem cell” (CSC). This CSC seems to drive and maintain cancer development [2,3,4,5]. Evidence for this new vision in cancer development was acquired for a variety of cancers, both leukemias [6,7,8,9,10,11,12,13] and solid cancers [14,15,16,17,18,19,20,21,22,23,24,25,26]. Under this new criterion, the CSCs are exclusively responsible for maintaining cancer development [27]. Thus, cancer development and maintenance are a result of modified CSC differentiation and not cellular proliferation [28,29], as postulated until recently. Under this new paradigm, our view of cancer development needs to be re-evaluated, particularly in what refers to the initial stages of cancer development [30,31].

Moving the maintenance of cancer development to the stem cell level has severe consequences for developing cancer therapies [2,3,4,5,32]. Traditional cancer therapy approaches tend to target the proliferating cancer cells, mostly leaving the CSC fraction unaffected. The initial cancer therapeutic success is usually followed by disease relapse as the remaining CSC pool repopulates the cancer [2,3,4,5,32]. Thus, new therapies need to be designed to target and remove this cellular source of the cancers and, when combined with conventional anti-proliferative therapies, will most likely be able to achieve cancer cure. The aim of this review is to present the current status of the field, naming if vitamin D analogues may allow an avenue to target the leukemic stem cells (LSCs) and to serve as a beginning for future studies.

## 2. The Modern Approach in Cancer Therapy

The use of chemotherapy as the main weapon for killing cancer cells traces back to the 1940s. Since then, the development of cancer drugs has become a multi-billion dollar industry. The main foundation behind cancer chemotherapy has relied on the biological property that the majority of the cells within a tumor are vigorously proliferating more than the normal cells within human tissues [1]. From this point of view, it is clear that these cytotoxic-based approaches (radiotherapy and/or chemotherapy) used in cancer treatment induced serious side effects, sometimes life-threatening, as a result of affecting normal cells with which cancer cells share many features. All current cancer anti-proliferative drugs owe their very limited clinical efficacy to the high toxicities against normal cells with high proliferation rates, such as the cells forming the blood, intestine, and hair follicles. These side effects associated with the development of drug resistance and clinical disease progression result in the probable failure of the therapy [1,4,32].

In the late 1980s, new attempts to develop more specific cytotoxic drugs led to the identification of signaling pathways specifically modified in cancer cells [1]. The new challenge was to develop new compounds that would specifically target the cancer cell molecular defect, thereby contributing to a more specific cancer therapy. The idea was that such cancer targeted therapies would selectively kill cancer cells without affecting normal cells. Thus, this modern approach would potentially reduce the side effects produced by classical anticancer approaches. This transition resulted in an important progress, but the main principles of cancer drug development and resistance remained the same to those used in the period from 1950 to 1980.

All these cancer therapy approaches (targeted or non-targeted) aim to kill proliferating cancer cells. By using this strategy, in spite of the huge investment, the average cancer survival in the last 35 years has been improved by 17% only [33]. The problem therefore still remains: how to develop precise and efficient cancer treatments?

## 3. A New Approach Aims to Target Cancer Stem Cell (CSCs)

The CSC theory about cancer development is a modernized version of the “embryonal rest hypothesis” formulated to explain the similarities between teratocarcinomas and an embryo more than 150 years ago [34,35]. The failure of current cancer therapeutic approaches in curing patients can be easily explained if we examine how the toxic effects produced by the therapies disappear once the treatment is discontinued. Those tissues requiring persistent self-renewal (for instance, hair, intestine, or hematopoietic tissue) are the most damaged by current antiproliferative cancer therapies. However, the normal function within these tissues is quickly reestablished once the cancer treatment is ceased. Cancer tissue is affected much the same by the antiproliferative cancer therapies. This would imply a similar level of cellular organization to cancer tissue to that of normal tissues in which a small fraction of stem cells are responsible for generating the tumor differentiated cells. The stem cells fraction within the tumor is slow-cycling and resistant to the antiproliferative cancer therapy. On the contrary, the main tumor mass, formed by the differentiated and proliferative cancer cells, responds to the antiproliferative cancer therapy. Based on the CSC theory of cancer development, we can concede that CSCs are resistant to chemotherapy by nature. As a result of this, CSCs can survive the cancer therapy and re-populate the cancer tissue [2,3,4,32]. Thus, the presence of CSCs implies the existence of a small fraction of slow-cycling cancer cells that are not killed by the anti-proliferative treatments, although this therapy is able to eliminate their cellular descendants. However, the abovementioned observations, mainly inferred from human targeted-therapy clinical failures, might suggest that cancer drivers have a non-homogenous mode of action throughout the cancer cell population [30,31,36,37]. This would point out the different human targeted-therapy clinical failures mentioned above. In this regard, there are novel *in vivo* genetic confirmations showing that human cancer drivers can direct stem cells (LSCs) towards precise and definite differentiated cancer cell fates. However, these oncogenes are not required by LSCs maintenance [38,39,40,41,42,43,44,45]. Consequently, this tumoral reprogramming phenomenon can be defined as the process by which a cancer driver can rearrange the epigenetic and/or transcriptome condition of the cancer cell-of-origin. As a result of this epigenetic reprogramming, a novel and precise pathological differentiation program is fixed resulting in cancer development [30,36]. The initiating cancer alteration would be the driving force in the epigenetic reprogramming process, essential for carcinogenesis. However, once reprogramming has been established this cancer driver would only be a passenger cancer alteration within the LSC. This mode of action of cancer drivers within LSCs explains why oncogene-based targeted therapies fail in removing the LSC fraction [38,43,46], in spite of their initial efficacy against the cancer differentiated cells.

## 4. Practical Implications of the Leukemic Stem Cells (LSCs) in Therapy

There is an increasing perception that LSCs represent an immense challenge to effective cancer treatment, as they are able to survive current clinical drugs. A key challenge for developing specific drugs against LSCs is to distinguish them from the normal stem cells. To this aim, the identification of unique LSC molecular targets is required. Thus, a deeper knowledge of both normal stem cell biology and LSC biology will be essential for naming such targets.

In the hematopoietic system the vitamin D pathway influences both cell differentiation and their final activation once differentiated. However, its relevance in various leukemia states remains poorly understood. Nevertheless, it is known that vitamin D promotes differentiation of myeloid differentiated cell under certain conditions, and it has been proposed that some types of myeloid leukemias (*i.e.*, Chronic myelomonocytic leukemia (CMML)) may benefit from vitamin D supplementation [47]. Similarly, the immune environment of patients with hematopoietic cancers can be modified by the immune-modulatory effects induced by vitamin D [47]. However, in order to use vitamin D analogues to target LSCs, we need to verify if the vitamin D receptor (VDR) might be used to distinguish LSCs from the normal counterparts.

## 5. Vitamin D Receptors (VDRs) on Hematopoietic Cells and LSCs

**Figure 1:**
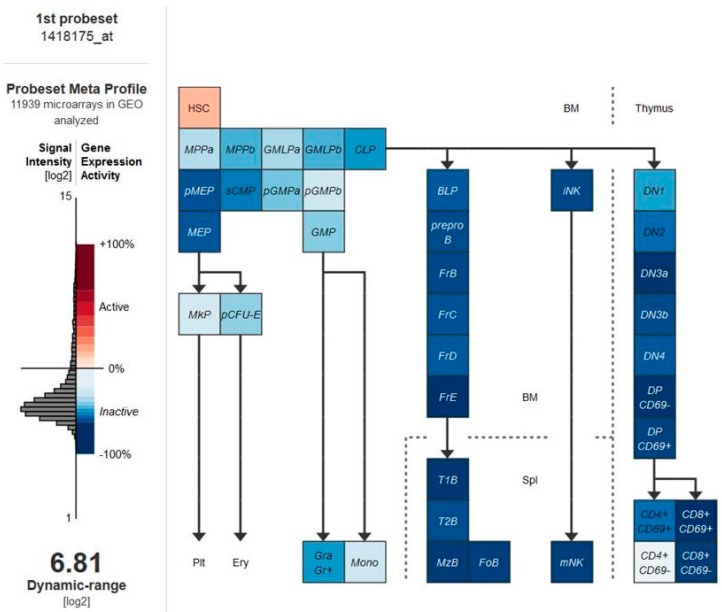
The vitamin D receptor (*VDR*) gene is significantly higher in normal hematopoietic stem cells (HSCs) than in the committed progenitor cells. We analyzed the relative expression of the *VDR* gene within 39 populations of mouse hematopoietic cells exploiting the Gene Expression Commons platform [48] (increased expression is represented in pink and decreased expression in blue). HSC (Hematopoietic Stem Cells population); MPPa (Multi-potent Progenitor subset A); MPPb (Multi-potent Progenitor subset B); GMLPa (Granulo/Macrophage/Lymphoid Progenitor subset A); GMLPb (Granulo/Macrophage/Lymphoid Progenitor subset B); CLP (Common Lymphoid Progenitor); pMEP (pre Megakaryocyte/Erythrocyte Progenitor); sCMP (Strict Common Myeloid Progenitor); pGMPa (preGranulocyte/Macrophage Progenitor subset A); pGMPb (preGranulocyte/Macrophage Progenitor subset B); MEP (Megakaryocyte/Erythrocyte Progenitor); GMP (Granulocyte/Macrophage Progenitor); MkP (Megakaryocyte Progenitor); pCFU-E (preCFU-E); Plt (Platelets); Ery (Erythrocyte); Gra (Granulocyte); Mono (Monocyte); BLP (Earliest B-lymphoid Progenitor); preproB (preproB cells); FrB (Fraction B B-cell); FrC (Fraction C B-cell); FrD (Fraction D B-cell); FrE (Fraction E B-cell); T1B (T1 B-cell); T2B (T2 B-cell); MzB (Marginal Zone B-cell); FoB (Folicular B-cell); iNK (intermediate Natural Killer Cell); mNK (mature Natural Killer Cell); DN (Double Negative T-cell); DP (Double Positive T-cell).

**Figure 2:**
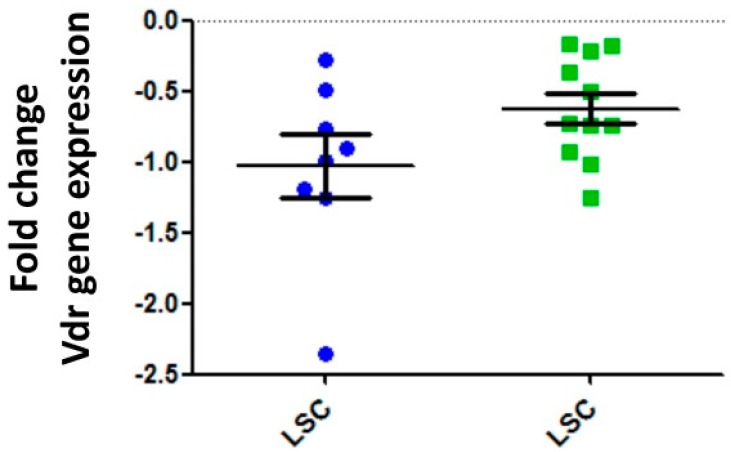
Leukemic stem cells (LSCs) share features with normal hematopoietic stem cells. Gene expression profiles of purified LSC populations were compared *versus* normal HSCs in mouse models expressing either MafB oncogene (blue dots) or Bcl6 oncogene (green dots). The same approach was used to isolate LSC and HSC (Sca1^+^Lin^-^ cells were purified). The ratios of the LSCs were referred to the control hematopoietic stem cells represented with the dotted line.

**Figure 3:**
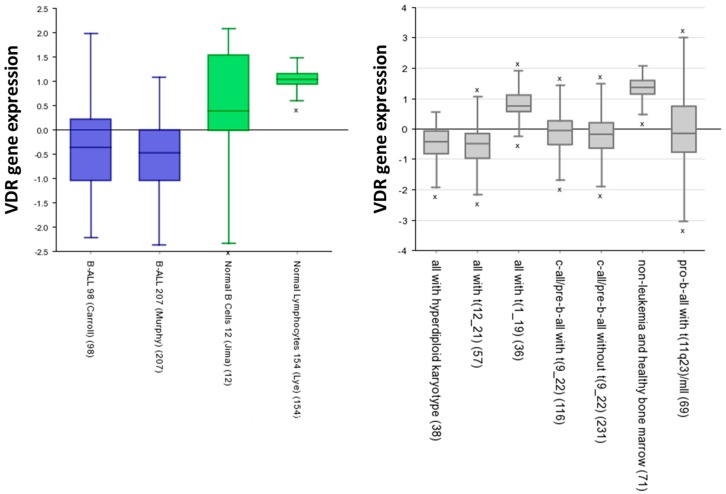
VDR expression is lower in human LSCs than in normal B cells. Using the R2 (http://r2.amc.nl) Genomics analysis and visualization platform [49] we analyzed *VDR* gene expression in human leukemic cells and in normal B cells from different datasets available in the platform. The expression values are represented in a transform z-score. The VDR Probeset used was 204254_s_at. “x” represents outlier.

**Figure 4:**
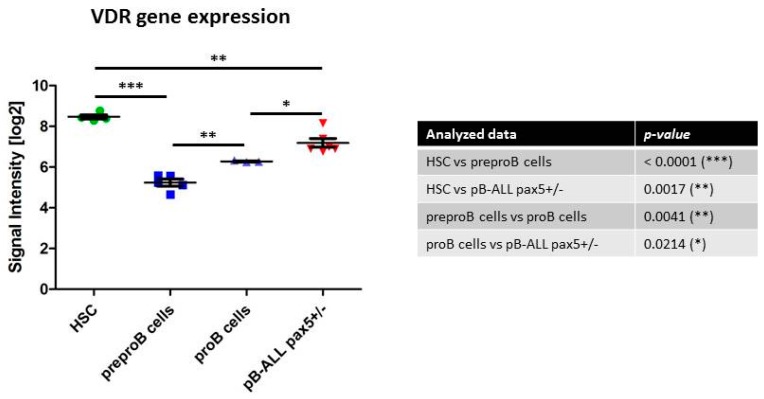
VDR (vitamin D receptor) expression in normal HSC, preproB cells, and proB cells subsets compared to pB-ALL cells from pax5+/− mice. Using publicly available gene expression microarray data [48], we compared VDR transcript abundance in normal murine HSC and preproB cells examined using *Affymetrix Mouse Genome 430 2.0 Array* with control pro-cells and pB-ALL cells from pax5+/− examined using *Affymetrix Mouse Gene 1.0 ST arrays* [50]. The highest expression of *VDR* gene was observed in normal HSC followed by the tumor differentiated cells from leukemic pax5+/− mice. Error bars represent the standard deviation. The Unpaired *t*-test has been used and *p*-values are indicated in the table.

In order to identify if VDRs are associated with LSCs, we initially analyzed VDRs’ expression within normal hematopoietic system. As illustrated in Figure 1, the expression levels of the vitamin D receptor gene are significantly higher in normal hematopoietic stem cells (HSCs) than in the committed progenitor cells [48]. We next proceeded to examine how the VDR expression in LSCs purified from different mouse models of hematopoietic cancers compared to control wild-type HSCs. The data identified that vitamin D receptor gene expression levels are significantly lower in LSCs than in normal HSCs (Figure 2). Similar results were observed when VDR expression was analyzed in human normal and leukemic samples (Figure 3). Thus, we next examined the expression of VDR in tumor differentiated cells *versus* normal counterparts. As illustrated in Figure 4, VDR expression is significantly higher in tumor differentiated cells than in the normal committed progenitor cells. Overall, these observations identify VDR as a potential attractive target for selective tumor differentiated cell eradication.

## 6. Vitamin D and the Epigenome

The pattern of expression of VDR in LSCs *versus* normal HSCs would not warrant an advisement to use it as a target to kill LSC. However, as we have mentioned before, LSCs are created as a result of a tumoral epigenetic reprogramming mechanism [30,36] and molecules able to modify the epigenetic status of LSCs could be used to modify their fate. In this regard, primary epigenetic effects of vitamin D seem to be mediated by histone modifications, mainly acetylation. Vitamin D-induced transcriptional activation is mediated by the VDR/RXR (retinoid X receptor) dimmer through interaction with histone acetyltransferases (HATs) [51]. VDR protein associates physically with coactivator and corepressor proteins, which in turn touch both chromatin modifiers, such as HATs, histone deacetylases (HDACs), histone methyltransferases (HMTs), and chromatin remodelers like histone demethylases (HDMs) of the Jumonji C (JmjC)-domain containing proteins and lysine-specific demethylase (LSD) families. Moreover, a number of genes encoding for chromatin modifiers and remodelers represent primary targets of VDR and its ligands. Finally, there is evidence that some VDR ligands possess DNA demethylating effects. In this regard, recent evidence associates DNA methylation changes in leukocytes to severe vitamin D deficiency, although the observed differences were not big [52]. Overall, these epigenetic events mediated by 1,25-D_3_ could be used to prevent or delay carcinogenesis by modifying the epigenetic status of LSCs.

## 7. Future Directions

Epidemiological studies support the notion that low serum vitamin D level is associated with an increased risk of a number of cancers. However, convincing evidence that vitamin D supplementation alters the risk of most cancers is lacking. Many preclinical studies, however, suggest that exposing cancer cells to pharmacological concentrations of active metabolites of vitamin D can induce differentiation, growth arrest, and eventually induce apoptosis. Consequently, high doses of Vitamin D (calcitriol) and several of its synthetic analogues have been shown to slow or stop the *in vivo* growth of many different tumors in animal models [53]. There are no data to support the notion that one type of cancer is more or less susceptible to the effects of vitamin D [54]. Despite examples of exceptional clinical efficacy, barriers remain to the successful use of vitamin D against many cancers. These include the lack of identification of particularly sensitive cancer target cell populations and the need to better comprehend the impact of vitamin D on the LSC epigenome. The findings presented here will hopefully lead to improved treatment options for vitamin D analogues in leukemia patients.

Finally, we should keep in mind when we try to develop therapies to target LSCs that the concept of LSC plasticity and bidirectional transformation between stem and non-stem cells has introduced additional difficulty to the complex process of intratumoral heterogeneity [55,56,57]. Clearly, this LSC plasticity may limit the effect of LSC-based treatments. Therapies targeting LSCs would only provide a temporal benefit by eliminating this population, since new LSCs might arise from non-LSCs left untouched. Therefore, it will be necessary to develop treatments against LSCs in each of their dynamic states in order to achieve a more complete cancer therapeutic responses, and ultimately to combine these specific LSC-based therapies with approaches targeting the bulk population in order to achieve a successful clinical response.

## References

[1] Etzioni R., Urban N., Ramsey S., McIntosh M., Schwartz S., Reid B., Radich J., Anderson G., Hartwell L. (2003). The case for early detection. Nat. Rev. Cancer.

[2] Dalerba P., Cho R.W., Clarke M.F. (2007). Cancer stem cells: Models and concepts. Annu. Rev. Med..

[3] Reya T., Morrison S.J., Clarke M.F., Weissman I.L. (2001). Stem cells, cancer, and cancer stem cells. Nature.

[4] Perez-Caro M., Sanchez-Garcia I. (2006). Killing time for cancer stem cells (CSC): Discovery and development of selective CSC inhibitors. Curr. Med. Chem..

[5] Chabner B.A., Roberts T.G. (2005). Timeline: Chemotherapy and the war on cancer. Nat. Rev. Cancer.

[6] Bonnet D., Dick J.E. (1997). Human acute myeloid leukemia is organized as a hierarchy that originates from a primitive hematopoietic cell. Nat. Med..

[7] Hope K.J., Jin L., Dick J.E. (2004). Acute myeloid leukemia originates from a hierarchy of leukemic stem cell classes that differ in self-renewal capacity. Nat. Immunol..

[8] Miyamoto T., Weissman I.L., Akashi K. (2000). AML1/ETO-expressing nonleukemic stem cells in acute myelogenous leukemia with 8;21 chromosomal translocation. Proc. Natl. Acad. Sci. USA.

[9] Cobaleda C., Gutierrez-Cianca N., Perez-Losada J., Flores T., Garcia-Sanz R., Gonzalez M., Sanchez-Garcia I. (2000). A primitive hematopoietic cell is the target for the leukemic transformation in human philadelphia-positive acute lymphoblastic leukemia. Blood.

[10] Cox C.V., Evely R.S., Oakhill A., Pamphilon D.H., Goulden N.J., Blair A. (2004). Characterization of acute lymphoblastic leukemia progenitor cells. Blood.

[11] Cox C.V., Martin H.M., Kearns P.R., Virgo P., Evely R.S., Blair A. (2007). Characterization of a progenitor cell population in childhood T-cell acute lymphoblastic leukemia. Blood.

[12] Ren R. (2005). Mechanisms of BCR-ABL in the pathogenesis of chronic myelogenous leukaemia. Nat. Rev. Cancer.

[13] Melo J.V., Barnes D.J. (2007). Chronic myeloid leukaemia as a model of disease evolution in human cancer. Nat. Rev. Cancer.

[14] Al-Hajj M., Wicha M.S., Benito-Hernandez A., Morrison S.J., Clarke M.F. (2003). Prospective identification of tumorigenic breast cancer cells. Proc. Natl. Acad. Sci. USA.

[15] Dick J.E. (2003). Breast cancer stem cells revealed. Proc. Natl. Acad. Sci. USA.

[16] Dalerba P., Dylla S.J., Park I.K., Liu R., Wang X., Cho R.W., Hoey T., Gurney A., Huang E.H., Simeone D.M. (2007). Phenotypic characterization of human colorectal cancer stem cells. Proc. Natl. Acad. Sci. USA.

[17] O’Brien C.A., Pollett A., Gallinger S., Dick J.E. (2007). A human colon cancer cell capable of initiating tumour growth in immunodeficient mice. Nature.

[18] Ricci-Vitiani L., Lombardi D.G., Pilozzi E., Biffoni M., Todaro M., Peschle C., de Maria R. (2007). Identification and expansion of human colon-cancer-initiating cells. Nature.

[19] Bao S., Wu Q., McLendon R.E., Hao Y., Shi Q., Hjelmeland A.B., Dewhirst M.W., Bigner D.D., Rich J.N. (2006). Glioma stem cells promote radioresistance by preferential activation of the DNA damage response. Nature.

[20] Bao S., Wu Q., Sathornsumetee S., Hao Y., Li Z., Hjelmeland A.B., Shi Q., McLendon R.E., Bigner D.D., Rich J.N. (2006). Stem cell-like glioma cells promote tumor angiogenesis through vascular endothelial growth factor. Cancer Res..

[21] Singh S.K., Hawkins C., Clarke I.D., Squire J.A., Bayani J., Hide T., Henkelman R.M., Cusimano M.D., Dirks P.B. (2004). Identification of human brain tumour initiating cells. Nature.

[22] Piccirillo S.G., Reynolds B.A., Zanetti N., Lamorte G., Binda E., Broggi G., Brem H., Olivi A., Dimeco F., Vescovi A.L. (2006). Bone morphogenetic proteins inhibit the tumorigenic potential of human brain tumour-initiating cells. Nature.

[23] Li C., Heidt D.G., Dalerba P., Burant C.F., Zhang L., Adsay V., Wicha M., Clarke M.F., Simeone D.M. (2007). Identification of pancreatic cancer stem cells. Cancer Res..

[24] Prince M.E., Sivanandan R., Kaczorowski A., Wolf G.T., Kaplan M.J., Dalerba P., Weissman I.L., Clarke M.F., Ailles L.E. (2007). Identification of a subpopulation of cells with cancer stem cell properties in head and neck squamous cell carcinoma. Proc. Natl. Acad. Sci. USA.

[25] Kim C.F., Jackson E.L., Woolfenden A.E., Lawrence S., Babar I., Vogel S., Crowley D., Bronson R.T., Jacks T. (2005). Identification of bronchioalveolar stem cells in normal lung and lung cancer. Cell.

[26] Collins A.T., Berry P.A., Hyde C., Stower M.J., Maitland N.J. (2005). Prospective identification of tumorigenic prostate cancer stem cells. Cancer Res..

[27] Cobaleda C., Sanchez-Garcia I. (2009). B-cell acute lymphoblastic leukaemia: Towards understanding its cellular origin. Bioessays.

[28] Brown G., Sanchez-Garcia I. (2015). Is lineage decision-making restricted during tumoral reprograming of haematopoietic stem cells?. Oncotarget.

[29] Sanchez-Garcia I. (2010). Getting to the stem of cancer. Semin Cancer Biol..

[30] Vicente-Duenas C., Hauer J., Ruiz-Roca L., Ingenhag D., Rodriguez-Meira A., Auer F., Borkhardt A., Sanchez-Garcia I. (2015). Tumoral stem cell reprogramming as a driver of cancer: Theory, biological models, implications in cancer therapy. Semin. Cancer Biol..

[31] Sanchez-Garcia I. (2015). How tumour cell identity is established?. Semin. Cancer Biol..

[32] Dean M., Fojo T., Bates S. (2005). Tumour stem cells and drug resistance. Nat. Rev. Cancer.

[33] Ries L.A.G., Harkins D., Krapcho M., Mariotto A., Miller B., Feuer E.J., Clegg L., Eisner M.P., Horner M.J., Howlader N. Seer Cancer Statistics Review, 1975–2003. http://seer.Cancer.Gov/csr/1975_2003/.

[34] Cohnheim J. (1867). Ueber entzundung und eiterung. Pathol. Anat. Physiol. Klin. Med..

[35] Virchow R. (1855). Virchows Arch. Pathol. Anat. Physiol. Klin. Med..

[36] Vicente-Duenas C., Romero-Camarero I., Cobaleda C., Sanchez-Garcia I. (2013). Function of oncogenes in cancer development: A changing paradigm. EMBO J..

[37] Martin-Lorenzo A., Gonzalez-Herrero I., Rodriguez-Hernandez G., Garcia-Ramirez I., Vicente-Duenas C., Sanchez-Garcia I. (2014). Early epigenetic cancer decisions. Biol. Chem..

[38] Perez-Caro M., Cobaleda C., Gonzalez-Herrero I., Vicente-Duenas C., Bermejo-Rodriguez C., Sanchez-Beato M., Orfao A., Pintado B., Flores T., Sanchez-Martin M. (2009). Cancer induction by restriction of oncogene expression to the stem cell compartment. EMBO J..

[39] Vicente-Duenas C., Perez-Caro M., Abollo-Jimenez F., Cobaleda C., Sanchez-Garcia I. (2009). Stem-cell driven cancer: “Hands-off” regulation of cancer development. Cell Cycle.

[40] Vicente-Duenas C., Gonzalez-Herrero I., Cenador M.B., Criado F.J., Sanchez-Garcia I. (2012). Loss of p53 exacerbates multiple myeloma phenotype by facilitating the reprogramming of hematopoietic stem/progenitor cells to malignant plasma cells by mafb. Cell Cycle.

[41] Vicente-Duenas C., Romero-Camarero I., Gonzalez-Herrero I., Alonso-Escudero E., Abollo-Jimenez F., Jiang X., Gutierrez N.C., Orfao A., Marin N., Villar L.M. (2012). A novel molecular mechanism involved in multiple myeloma development revealed by targeting mafb to haematopoietic progenitors. EMBO J..

[42] Vicente-Duenas C., Fontan L., Gonzalez-Herrero I., Romero-Camarero I., Segura V., Aznar M.A., Alonso-Escudero E., Campos-Sanchez E., Ruiz-Roca L., Barajas-Diego M. (2012). Expression of MALT1 oncogene in hematopoietic stem/progenitor cells recapitulates the pathogenesis of human lymphoma in mice. Proc. Natl. Acad. Sci. USA.

[43] Velasco-Hernandez T., Vicente-Duenas C., Sanchez-Garcia I., Martin-Zanca D. (2012). P53 restoration kills primitive leukemia cells in vivo and increases survival of leukemic mice. Cell Cycle.

[44] Romero-Camarero I., Jiang X., Natkunam Y., Lu X., Vicente-Duenas C., Gonzalez-Herrero I., Flores T., Luis Garcia J., McNamara G., Kunder C. (2013). Germinal centre protein HGAL promotes lymphoid hyperplasia and amyloidosis via BCR-mediated Syk activation. Nat. Commun..

[45] Green M.R., Vicente-Duenas C., Romero-Camarero I., Long Liu C., Dai B., Gonzalez-Herrero I., Garcia-Ramirez I., Alonso-Escudero E., Iqbal J., Chan W.C. (2014). Transient expression of Bcl6 is sufficient for oncogenic function and induction of mature B-cell lymphoma. Nat. Commun..

[46] Prost S., Relouzat F., Spentchian M., Ouzegdouh Y., Saliba J., Massonnet G., Beressi J.P., Verhoeyen E., Raggueneau V., Maneglier B. (2015). Erosion of the chronic myeloid leukaemia stem cell pool by PPARγ agonists. Nature.

[47] Hall A.C., Juckett M.B. (2013). The role of vitamin D in hematologic disease and stem cell transplantation. Nutrients.

[48] Seita J., Sahoo D., Rossi D.J., Bhattacharya D., Serwold T., Inlay M.A., Ehrlich L.I., Fathman J.W., Dill D.L., Weissman I.L. (2012). Gene expression commons: An open platform for absolute gene expression profiling. PLoS ONE.

[49] R2: Genomics analysis and visualization platform. http://r2.Amc.nl.

[50] Martin-Lorenzo A., Hauer J., Vicente-Duenas C., Auer F., Gonzalez-Herrero I., Garcia-Ramirez I., Ginzel S., Thiele R., Constantinescu S.N., Bartenhagen C. (2015). Infection exposure is a causal factor in b-cell precursor acute lymphoblastic leukemia as a result of pax5-inherited susceptibility. Cancer Discov..

[51] Karlic H., Varga F. (2011). Impact of vitamin D metabolism on clinical epigenetics. Clin. Epigenet..

[52] Zhu H., Wang X., Shi H., Su S., Harshfield G.A., Gutin B., Snieder H., Dong Y. (2013). A genome-wide methylation study of severe vitamin D deficiency in african american adolescents. J. Pediatr..

[53] Hughes P.J., Marcinkowska E., Gocek E., Studzinski G.P., Brown G. (2010). Vitamin D_3_-driven signals for myeloid cell differentiation--Implications for differentiation therapy. Leuk. Res..

[54] Krishnan A.V., Trump D.L., Johnson C.S., Feldman D. (2012). The role of vitamin D in cancer prevention and treatment. Rheum. Dis. Clin. N. Am..

[55] Doherty M.R., Smigiel J.M., Junk D.J., Jackson M.W. (2016). Cancer stem cell plasticity drives therapeutic resistance. Cancers (Basel).

[56] Meacham C.E., Morrison S.J. (2013). Tumour heterogeneity and cancer cell plasticity. Nature.

[57] Tang D.G. (2012). Understanding cancer stem cell heterogeneity and plasticity. Cell Res..

